# Effect of Data Quality and Data Quantity on the Estimation of Intrinsic Solubility: Analysis Based on a Single-Source Data Set

**DOI:** 10.1021/acs.molpharmaceut.4c00685

**Published:** 2024-09-13

**Authors:** Jiaxi Zhao, Eline Hermans, Kia Sepassi, Christophe Tistaert, Christel A. S. Bergström, Mazen Ahmad, Per Larsson

**Affiliations:** † Department of Pharmacy, Uppsala University, 751 23 Uppsala, Sweden; ‡ Pharmaceutical & Material Sciences, Janssen Pharmaceutica NV, B-2340 Beerse, Belgium; § Discovery Pharmaceutics, Janssen Research & Development, LLC, La Jolla, California 92121, United States; ∥ In Silico Discovery, Janssen Pharmaceutica NV, B-2340 Beerse, Belgium

**Keywords:** solubility prediction, machine learning, quantitative structure−property relationship (QSPR), intrinsic solubility, data quality

## Abstract

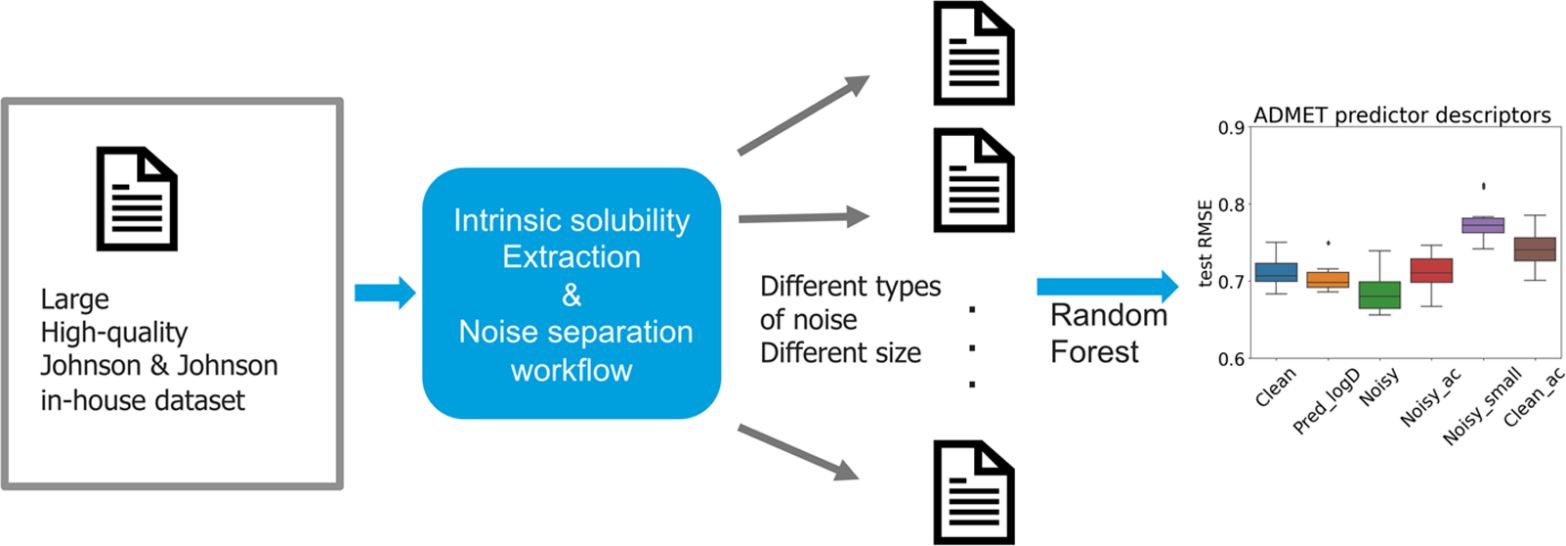

Aqueous solubility is one of the most important physicochemical properties of drug molecules and a major driving force for oral drug absorption. To date, the performance of in silico models for the estimation of solubility for novel chemical space is limited. To investigate possible reasons and remedies for this, the Johnson and Johnson in-house aqueous solubility data with over 40,000 compounds was leveraged. All data were generated through the same high-throughput assay, providing a unique opportunity to explore the relationship between data quality, quantity, and model estimations. Six intrinsic solubility data sets with different sizes and noise levels were generated by making use of three different approaches: (i) inclusion or exclusion of amorphous solid residue, (ii) measured or experimental log *D* to identify the intrinsic solubility, and (iii) adopting or omitting a quality check process in the data processing workflow. A random forest regressor was trained on the data sets with three different sets of descriptors calculated from RDKit, ADMET predictor, or Mordred, and the performances were evaluated with nested cross-validation as well as ten refined test sets. The models confirm, as expected, that with the same data set size, high-quality data leads to better model performance; however, also, models trained with larger data sets containing analytical variability can give equally accurate estimations compared to models trained with small, clean, and diverse data sets. However, noise introduced by including the presence of amorphous solid postsolubility measurement in the training data set cannot be overcome by increasing data size, as they are introducing a biased systematic positive error in the data set, confirming the importance of critical data review. Finally, two top-performing models were tested on the first test set from the second solubility challenge, achieving RMSE values of 0.74 and 0.72 and log *S* ± 0.5 of 46 and 48%, respectively. These results demonstrated improved performance compared to those reported in the findings of the competition, highlighting that a single-source curated data set can enhance the prediction of intrinsic solubility.

## Introduction

Solubility is an important physicochemical property of drug molecules because of the role of dissolution in the gastrointestinal tract for oral drug absorption. In particular, intrinsic solubility is defined as the aqueous solubility of a molecule in its uncharged state.^1^ Poorly soluble drugs can have poor bioavailability and consequently lack the pharmacological effect.^2,3^ To save time and cost in the development of new drugs, the identification of problematic compounds is of great importance early in drug discovery. However, the use of experimental assays for this purpose is restricted by the limited amounts of active pharmaceutical ingredients (APIs) synthesized in discovery. Also, the experiments can be expensive and time-consuming due to the large number of molecules being screened. Ideally, it would be great to already gain solubility information at chemistry ideation to assist in prioritizing compounds for synthesis. Hence, a quick and accurate way to obtain a first solubility estimate through in silico models is of high added value.

To this end, numerous computational methods to predict solubility based on the molecular structure have been developed (see, e.g., Bergström and Larsson^4^ for a more comprehensive overview). Approaches based on physical principles, such as molecular dynamics simulations, can, in principle, be used to calculate solubilities without experimental data. Still, the practical use of such methods is often hampered by the need for lengthy and resource-intensive calculations.^4,5^ Alternatives include methods based on equations of state (EOS) such as the Perturbed-chain SAFT (PC-SAFT),^6^ NRTL-SAC,^8^ or UNIFAC^9^ methods. One potential issue with these methods is that they, at least to some degree, rely on compound-specific experimental data for parametrization,^7^ which might not always be available and also might make it difficult for models to be generalizable.^10,11^ The Conductor-like screening model (COSMO) is also widely used for solubility predictions and can be particularly useful for solubility calculations in nonaqueous systems or in the presence of other excipients.^5^ Finally, different models based on the general solubility equation (GSE)^6^ have been developed, such as the Avdeef–Kansy “Flexible-Acceptor” General Solubility Equation (GSE(Φ,B)).^7^ A limitation of this particular type of method is the lack of melting point measurements in early discovery.

In pharmaceutical sciences, machine learning has been effectively used for molecule ideation,^8^ property prediction,^9^ chemical reaction optimization,^10,11^ virtual screening,^12^ drug formulation development,^13−15^ pharmaceutical manufacturing,^16^ etc. Its use specifically for solubility estimation started decades ago and initially used shallow machine learning algorithms such as random forest or support vector machine. In Avdeef’s work, a random forest regressor trained on data set Wiki-pS0 outperformed both the GSE and the Abraham solvation equation,^17^ with later studies revealing better performance with consensus models.^18−20^ With the emergence of more data and powerful computing abilities, more sophisticated models have been developed. Different architectures have been applied to predict solubilities, such as fully connected neural networks, recurrent neural networks,^21^ transformers,^22^ and graph neural networks.^23^ Over the years, various molecule representations have been investigated, including group contribution methods, fingerprints such as Extended-connectivity fingerprint^25^ (ECFP), two-dimensional (2D) molecular descriptors such as molecular weight, log *P*-values, and molecular graphs representing atoms as vertexes and bonds as edges. Different combinations of algorithms and molecule representations have also been tested.^24,25^ The first solubility challenge,^2^ introduced in 2008, and the second^26^ in 2019 were set up to assess and advance the state-of-the-art of solubility predictions. It has been argued that there is room for improvement within the models themselves.^27^ However, one conclusion that emerged was that accurate solubility prediction might be hampered by the lack of high-quality data in sufficient amounts.

Despite numerous attempts, the prediction accuracy of intrinsic solubility remains challenging. Many publications train models on data sets from multiple publicly available resources that lack reliable and reproducible solubility measurements or contain nondrug-like molecules. Apart from the inherent difficulty in solubility measurements, variations in experimental conditions can lead to large differences in the measured solubility value. Other factors that contribute to the introduction of errors and noise are the lack of pH measurements, experimental temperature, and the solid-state nature of the solid postsolubility measurement in some public data sets. Combining several data sets to increase the amount of training data then hampers the quality of the ultimate data set. For example, the data set from Benet et al. contains 900 solubility values, making it relatively large. However, for the compounds listed, not all of the compounds are accompanied by a measured solubility. For those with a reported solubility, it is the lowest measured/reported value in the literature across the pH 1–7.5 range. In addition, not all details are shared on the process and how different experimental assays were critically reviewed, for example, solid-state analysis, temperature, or equilibration time.

The second solubility challenge confirmed the need for larger and better databases generated under “good practice” with detailed experimental information. It also pointed out that future improvements can be achieved by using a single, critically curated intrinsic solubility data set with compounds that cover the chemical space of drugs. With guaranteed data quality, it will be easier to recognize significant improvements in prediction methodologies and guide molecule representation selection. The large, high-quality Johnson and Johnson (J&J) in-house discovery solubility data meets these requirements, as it was generated from the same assay. The data provides a solid foundation for studying the impact of data quality and quantity on aqueous intrinsic solubility prediction. Using this data, a new investigation method was developed that included a carefully constructed data processing workflow and the generation of six data sets with different types/levels of noise and size. Two evaluation experiments were conducted, utilizing nested cross-validation and 10 clean test sets, respectively, to evaluate the impact of size and quality by including random analytical variability and systematic noise introduced by amorphous compounds in the training set.

## Methods

### Data Sets

The two J&J in-house discovery pH 2 and pH 7 solubility data sets were used in our study. Both data sets contain the same structurally drug-like compounds (around 40,000 in each) with solubility (72 h time point) measured at pH 2 and 7, under agitation at 25 °C with high-throughput screening. The suspended, postsolubility solid state has been analyzed by polarized light microscopy to characterize it as either amorphous or crystalline.

To determine if our resulting data were indeed the intrinsic solubility, a J&J in-house log *D* data set was used. It contains chromatographically measured log *D* values at pH values of 2.6, 7.4, and 10.5. We used these values to identify the intrinsic solubility between pH 2 to pH 7 from both solubility data sets. Note that the log *D* measurements are not available for all compounds. Hence, we also used predicted log *D* values via the ADMET predictor (version 9.0) as an alternative (details below).

### Data Processing Workflow

**Figure 1:**
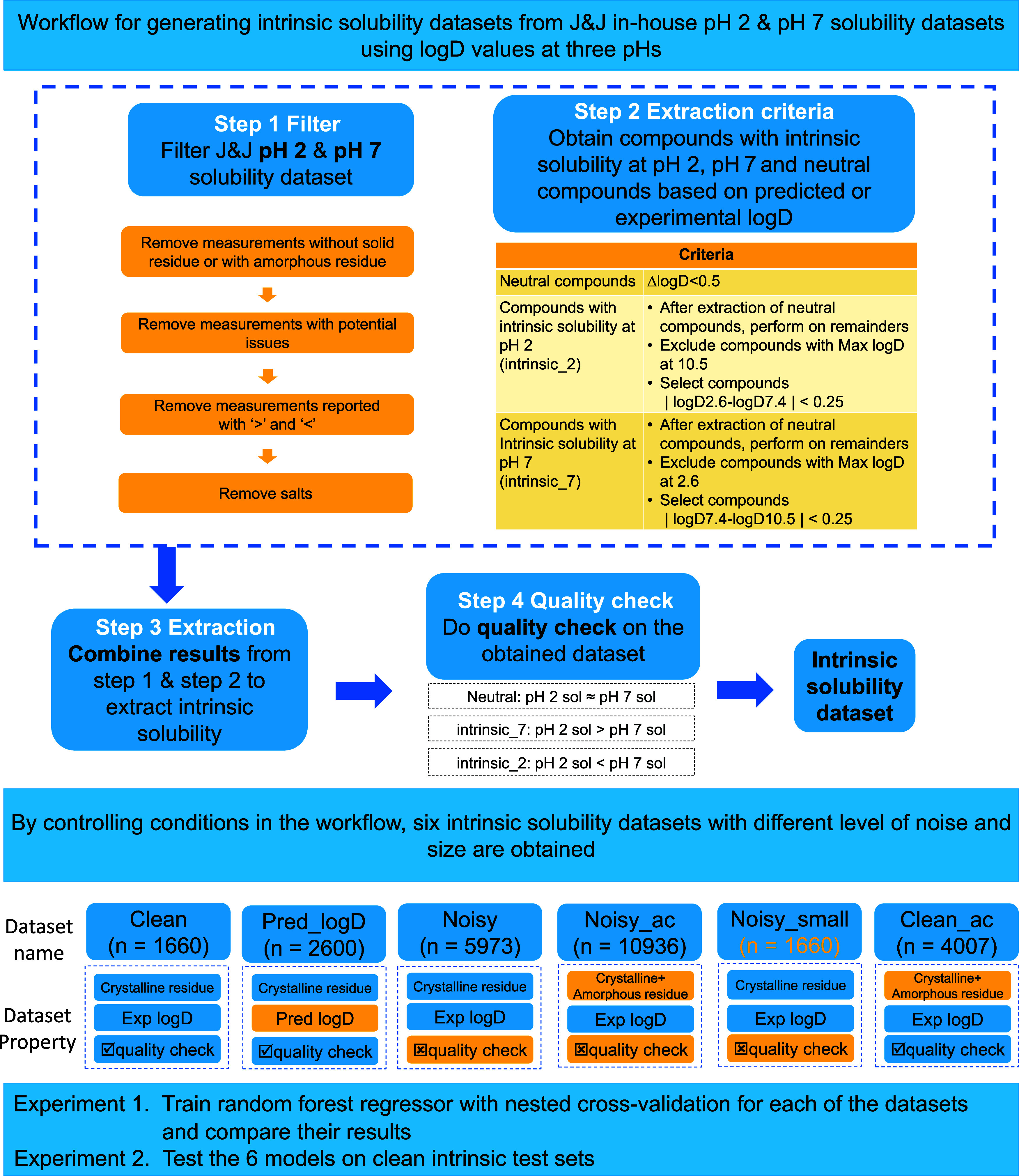
Data processing workflow for the generation of the six quality-controlled data sets. In the Data Property section, a blue cell represents the same generation condition as for **Clean**, while a yellow cell represents a different one.

The data processing workflow that we designed for this study encompassed three steps (Figure 1). First, a filtering step was executed on the pH and pH 7 solubility data sets. Second, an extraction step derived intrinsic solubility from the filtered data sets. Third, a quality check step was conducted on the extracted intrinsic solubility data set to minimize analytical variability.

For the filtering step of the two pH-dependent data sets, data points were removed for amorphous solid forms and when no solid residue was found in the postsolubility measurement. Due to the dynamic range of the solubility assay being within 0.1–600 μM, solubility values reported with “>” and “<” were also removed. Next, the salt forms of compounds were filtered out to focus the models on the free forms. Finally, stereoisomers were removed as the prediction of their solubility would have presented a complexity outside the scope of this study.

Since the focus of our study was the estimation of intrinsic solubility, additional steps were taken to infer it from the two pH-dependent solubility data sets. In the presence of ionization within a compound, log *D* decreases compared to its neutral form or, equivalently, log *D* reaches its maximum value when the compound is neutral. Consequently, log *D* values at pH values of 2.6, 7.4, and 10.5 were used to identify the pH at which the molecule is expected to be neutral. Log *D* values are used instead of p*K*_a_ values because experimental p*K*_a_ values are not available for most of the drug molecules, and the predicted p*K*_a_ values are considered less reliable than measured log *D* values. Compounds were considered neutral within pH 2.6–10.5 if the absolute difference among the three log *D* values was less than 0.5.

After the neutral compounds were identified and extracted, compounds with intrinsic solubility at pH 2 were extracted from the remaining compounds. First, we filtered out those with a maximum log *D* at pH 10.5. The remaining compounds that satisfied the criterion |log *D*_pH2.6_ – log *D*_pH7.4_| < 0.25 were selected. The extraction of compounds with intrinsic solubility at pH 7 followed a similar approach. Initially, compounds with maximum log *D* at 2.6 were excluded, and then compounds with |log *D*_pH7.4_ – log *D*_pH10.5_| < 0.25 were selected. The neutral compounds and compounds with intrinsic solubility at pH 2 and pH 7 resulted in an intrinsic solubility data set. These extraction criteria focus on the identification of the intrinsic solubility of exclusively acidic or basic molecules, excluding amphoteric molecules (in the pH range of 2.6–10.5), which may be a limitation of this study. Also, experimental log *D* values are not available for all compounds. Therefore, some compounds present in the discovery solubility database with intrinsic solubility at pH 2 or 7 may be missing in the obtained intrinsic solubility data set.

Finally, to minimize the analytical variability in the data set, we implemented a data quality check step. Neutral compounds do not display pH-dependent solubility at pH 2 and 7, so theoretically, their solubility should be identical at these two pH values. Thus, neutral compounds with greater than a 3-fold solubility difference at pH 2 and pH 7 were discarded. For compounds with an intrinsic solubility at pH 7, instances where the reported solubility values at pH 2 were 3-fold less than those at pH 7 were filtered out. Similarly, for compounds with intrinsic solubility at pH 2, cases where the solubility at pH 7 was 3-fold less than that at pH 2 were also filtered out. This final quality check led to a clean data set of intrinsic solubility measurements.

### Data Set Generation

The data processing workflow described above (i) excluded amorphous solid forms in the filtering step, (ii) used experimental log *D* to extract intrinsic solubility, and (iii) used a final quality check. We refer to the resulting data set with crystalline intrinsic solubility measurements as **Clean**.

- **Data set Clean (*n*** = **1660)** is the smallest but most refined data set compared to the other data sets generated later.

These strict criteria greatly reduced the number of eligible compounds selected from the 40,000 in the J&J solubility data. We therefore considered increasing the size of the data set at the expense of adding uncertainties or noise. This was done by either (i) including or excluding amorphous residue in the filtering step, (ii) using predicted log *D* values in the extraction step (as these can be calculated for all compounds), or (iii) adapting or ignoring the quality check process in the workflow. In this way, the following five intrinsic solubility data sets were generated.

- **Data set Pred_logD (*n*** = **2600)** was generated using predicted log *D* values from the ADMET predictor. The other extraction criteria were the same as for the **Clean** data set. This data set enabled analysis of whether predicted log *D* values could substitute for experimentally derived ones. This data set was larger than **Clean** since, again, not all compounds in the solubility database had experimental log *D* measurements. To assess the accuracy of the log *D* prediction, the predicted values for some 1650 out of the 2600 compounds for which experimental values existed were compared. The RMSE was found to be 0.17 for the log *D* predictions at pH 2.6, 0.15 at pH 7.4, and 0.15 at pH 10.5. Across all three pH values, the Pearson correlation coefficient was consistently 0.98.
- **Data set Noisy (*n*** = **5973)** omitted the final quality check step in **Clean**, giving 4313 additional compounds.
- **Data set Noisy_small (*n*** = **1660)** is the same size as the **Clean** data set but incorporates the same type and fraction of noise as **Noisy**. This enabled testing the impact of noisy data on model quality, irrespective of data set size.
- **Data set Clean_ac (*n*** = **4007)** includes data points obtained as a combination of both crystalline residue and amorphous residue in the data set. Other conditions were the same as for **Clean**. In general, amorphicity increases solubility and thus introduces a one-sided bias. However, we included it because we have observed that it is often encountered in discovery solubility databases.
- **Data set Noisy_ac (*n*** = **10,936)** contains data points combining both crystalline and amorphous residues. Other conditions were the same as for **Noisy**. This made **Noisy_ac** the largest of the generated data sets. It contained both random analytical variability (by omitting the final quality check) and biased noise (by including amorphous compounds). The difference in the number of compounds between the J&J data (40,000 compounds) and **Noisy_ac** comes from the filtering step and the exclusion of compounds with intrinsic solubility outside the range of pH 2 to 7.

To obtain a visual overview of the six data sets and the chemical space covered in each, we used UMAP^28^ to reduce the ECFP fingerprints and visualized them with 2D density plots. For comparing the chemical space, we used the Estimated SOLubility (ESOL) data set,^29^ containing 2874 compounds.

### Molecular Descriptors

Three molecular descriptor sets were calculated with RDKit,^30^ ADMET Predictor, or Mordred^31^ for each of the six data sets. In this way, both open-source and commercially available software were included in the study, as well as different numbers and types of descriptors (see the Supporting Information for details). To avoid multicollinearity, the Pearson correlation coefficients of each descriptor pair within each descriptor set were calculated. For pairs with a correlation greater than 0.9, only the descriptor with the higher correlation with solubility was kept for training the model. This resulted in 18 descriptors for the RDKit set, ∼260 for the ADMET predictors set, and ∼600 for the Mordred set.

### Model Selection

Following the creation of **Clean**, six machine-learning models were trained using it and compared with each other. These were: least absolute shrinkage and selection operator (LASSO), random forest regressor (RF), support vector regression (SVR), light gradient-boosting machine (LightGBM), Extreme Gradient Boosting (XGboost), and an artificial neural network (ANN). The model comparison was done using 18 RDKit descriptors. Compounds in **Clean** were randomly split into 80% training set and 20% test set. A 10-fold cross-validation based on the training set was used to evaluate and compare the performance of the models using the root mean squared error (RMSE) and coefficient of determination (*R*^2^) as evaluation metrics. Another metric, the percentage of compounds correctly predicted within 0.7 log units (% log *S* ± 0.7), was introduced as an orthogonal method for checking the accuracy of the model.^24^ A 0.7 log unit corresponds to a 5-fold difference in molar solubility, which is a typical and acceptable error in discovery solubility in vitro assays. Hyperparameter tuning was implemented using Optuna^32^ (version 3.5.0) (Supporting Information).

### Model Performance Evaluation Experiments Based on the Six Generated Data Sets

Following the identification of the RF as the optimal algorithm (details in the Results section) in this study, two experiments were performed with RF to evaluate the impact of data quality and quantity on intrinsic solubility prediction using the six generated data sets.

In the first evaluation experiment, random forest regressors were trained and evaluated via nested cross-validation (10-fold in the outer loop and 5-fold in the inner loop).

In the second evaluation experiment, ten clean intrinsic solubility data sets (*n* = 332) were randomly split (20%) from **Clean** and used as test data sets. Compounds in the test set(s) were removed from each of the six data sets, and random forest regression models were then trained on the remaining compounds. Performance was evaluated via the average metric value of the clean test sets. Finally, a one-way ANOVA and post hoc Tukey tests were performed over the RMSE values on the 6 data sets for each descriptor set to check for statistical significance (*p* < 0.05). Scipy^33^ package (version 1.8.1) was used for the statistical tests.

### Model Interpretation

For each descriptor set (RDKit, ADMET Predictor, Mordred), SHAP^34^ version 0.44.1 analysis was conducted on the models trained with **Clean** using the Python package TreeExplainer.^35^ Force plots (Figure S2) were produced to visualize the feature importance and impact on the solubility predictions.

## Results

Six intrinsic solubility data sets with different sizes and amounts of noise were generated from the J&J in-house discovery solubility database (Figure 1). Six machine-learning models were then trained on **Clean** with 18 RDKit descriptors as input, and the performances were evaluated with cross-validation. The best-performing model was then used in two comparison experiments to evaluate the impact of data quality and quantity on the intrinsic solubility prediction.

### Data Exploration

**Figure 2:**
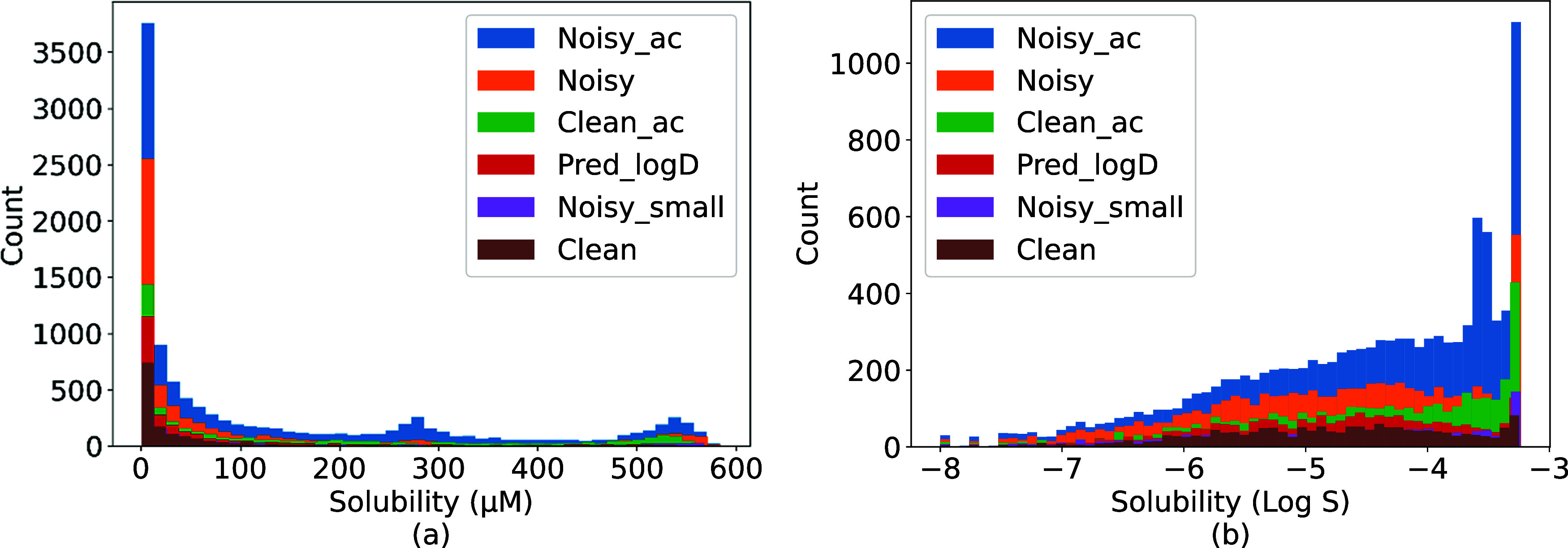
Solubility distribution of the six generated intrinsic solubility data sets. (a) μM unit and (b) logarithm unit.

Figure 2a shows the distribution of solubility values in each of the six data sets. All data sets are highly right-skewed, with most compounds having a reported solubility value between 0 and 100 μM (about 60–75% of compounds in all six data sets; additional details can be found in Figure S1). After changing the scale from μM to M, followed by a log 10 transformation, the solubility values were represented as Log *S* (Figure 2b).

**Figure 3:**
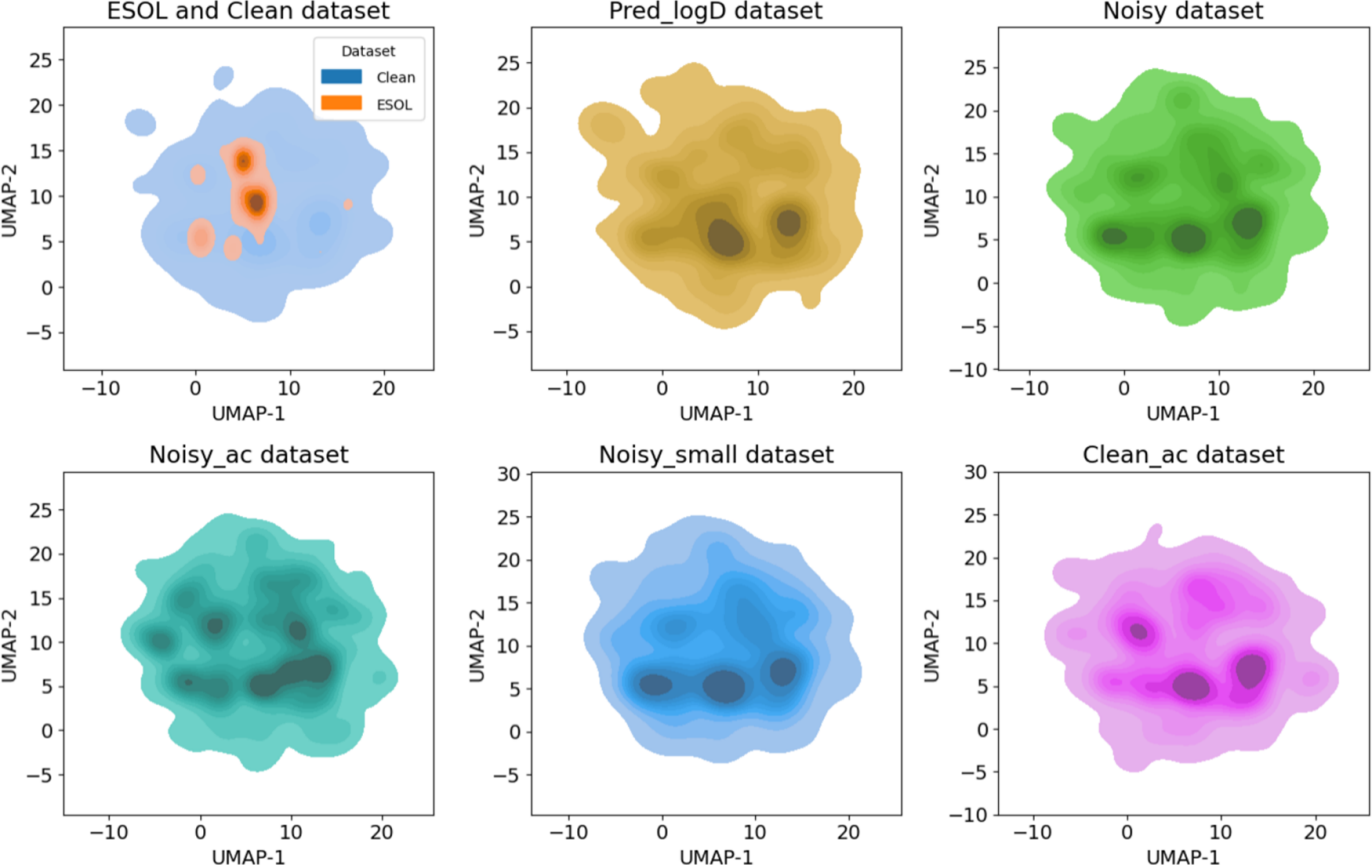
2D UMAP density plots of the chemical space of the six intrinsic solubility data sets. The ESOL data set (orange, upper left panel) is included for comparison.

The chemical spaces of the six generated data sets were plotted using UMAP, together with a similar analysis for the ESOL data set (Figure 3). Even though the data set sizes span from *n* = 1660 to *n* = 10,936, the generated data sets all covered similar regions in the chemical space. The ESOL data set contains more compounds (*n* = 2874) than **Clean**, but the chemical space of the latter is much larger (Figure 3, top left panel), reflecting the diversity of compounds in the data set.

### Model Selection

Six machine learning models were trained and compared to each other using **Clean** (Table 1). LASSO, SVR, and LightGBM had the highest bias, while the remaining models suffered from high variance. The RMSE values were in line with values reported in the literature (RMSE ∼0.7–1.1 log units^36^). The *R*^2^ values were also similar to those recently reported from the analysis of the second solubility challenge.^36,37^ The random forest algorithm was the optimal model with the lowest cross-validation RMSE and highest cross-validation *R*^2^, and it was therefore used throughout the rest of the experiments.

**Table 1:** RMSE and *R*^2^ of the Six Different Algorithms Trained on **Clean**

estimator	train RMSE	train *R*^2^	test RMSE	test *R*^2^	cross-validation RMSE	cross-validation *R*^2^	%log *S* ± 0.7
Lasso	0.83	0.30	0.82	0.29	0.84	0.28	60.5
**RF**	**0.37**	**0.86**	**0.76**	**0.40**	**0.77**	**0.39**	**67.8**
SVR	0.64	0.58	0.83	0.28	0.79	0.36	65.1
XGBoost	0.35	0.88	0.77	0.38	0.77	0.39	66.6
LightGBM	0.61	0.63	0.79	0.35	0.81	0.33	65.3
ANN	0.52	0.73	0.87	0.20	0.82	0.31	65.7

Note that in the following sections, the data set names are used as names for the corresponding trained model.

### Comparison of Model Performance Based on Nested Cross-Validation

Following the identification of the RF model as the best of the six algorithms, we assessed the impact of the descriptors on its performance. The RMSE and *R*^2^ metrics of the inner loop and outer loop were compared with each other (Table 2). Models trained with ADMET predictor descriptors performed best, followed in rank order by Mordred descriptors and RDKit. Based on the % log *S* ± 0.7 metric, the ADMET predictor was the best, while Mordred and RDKit were comparable to each other.

**Table 2:** Inner and Outer Loop RMSE and *R*^2^ for the RDKit, ADMET, and Mordred Descriptor Set^a^

RDKit	Clean	Pred_logD	Clean_ac	Noisy	Noisy_ac	Noisy_small
Inner Loop						
RMSE	0.78(0.01)	0.78(0.01)	0.85(0.0)	0.90(0.01)	0.89(0.00)	0.98(0.01)
*R*^2^	0.38(0.01)	0.38(0.01)	0.36(0.01)	0.31(0.01)	0.32(0.00)	0.21(0.01)
Outer Loop						
RMSE	0.76(0.04)	0.78(0.04)	0.83(0.03)	0.89(0.03)	0.88(0.01)	0.97(0.03)
*R*^2^	0.40(0.06)	0.39(0.03)	0.38 (0.03)	0.33(0.03)	0.34(0.02)	0.22(0.05)
%log *S* ± 0.7	66.6(2.0)	65.4(4.2)	63.3(1.4)	59.8(1.2)	60.8(1.2)	52.4(3.1)

ADMET	Clean	Pred_logD	Clean_ac	Noisy	Noisy_ac	Noisy_small
Inner Loop						
RMSE	0.74(0.01)	0.75(0.01)	0.80(0.00)	0.85(0.01)	0.83(0.00)	0.95(0.01)
*R*^2^	0.44(0.01)	0.43(0.01)	0.43(0.01)	0.39(0.01)	0.41(0.00)	0.25(0.01)
Outer Loop						
RMSE	0.72(0.05)	0.73(0.04)	0.78(0.03)	0.84(0.03)	0.81(0.01)	0.95(0.02)
*R*^2^	0.46(0.07)	0.46(0.03)	0.46 (0.04)	0.40(0.03)	0.43(0.02)	0.26(0.04)
%log *S* ± 0.7	67.9(2.5)	67.2(3.7)	64.9(2.3)	62.1(1.6)	63.8(1.3)	52.6(3.0)

Mordred	Clean	Pred_logD	Clean_ac	Noisy	Noisy_ac	Noisy_small
Inner Loop						
RMSE	0.76(0.01)	0.77(0.01)	0.82(0.01)	0.88(0.00)	0.87(0.00)	0.97(0.00)
*R*^2^	0.40(0.01)	0.40(0.01)	0.40(0.01)	0.34(0.00)	0.35(0.00)	0.22(0.00)
Outer Loop						
RMSE	0.75(0.04)	0.76 (0.04)	0.81(0.03)	0.87(0.03)	0.85(0.01)	0.97(0.02)
*R*^2^	0.42(0.06)	0.41(0.02)	0.42(0.04)	0.36(0.03)	0.38(0.02)	0.23(0.03)
%log *S* ± 0.7	65.6(3.6)	65.2(3.8)	63.3(2.3)	59.7(1.8)	61.1(1.4)	50.4(2.3)

a Values in parentheses are standard deviations.

**Figure 4:**
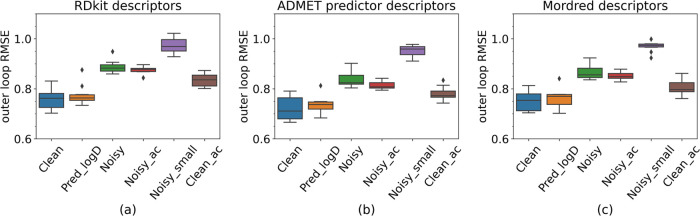
Outer loop RMSE for the descriptor sets: (a) RDKit, (b) ADMET, and (c) Mordred.

Next, the impact of different training sets on the model quality was assessed. A consistent underlying pattern could be seen across all three descriptor sets (Table 2 and box plots in Figure 4). The model trained with **Clean** delivered the best performance, and the **Pred_logD model** follows closely with similar results, followed in turn by the **Clean_ac, Noisy_ac**, and N**oisy** models. The **Noisy_small** model performed worst.

At first glance, these results aligned with our expectations: models trained with data sets of a lower quality or higher uncertainty tended to perform worse, even if the data set size was much larger. However, when the same high-quality test set was applied to all models, the outcomes were different, as discussed below.

### Comparison of Model Performance Based on Ten Refined Intrinsic Test Sets

In this second experiment, ten test sets, split out from **Clean**, were used to evaluate the models trained with the six data sets. Based on the averaged evaluation metrics RMSE and *R*^2^, again, the model trained with the ADMET predictor performed best and RDKit performed worst.

**Figure 5:**
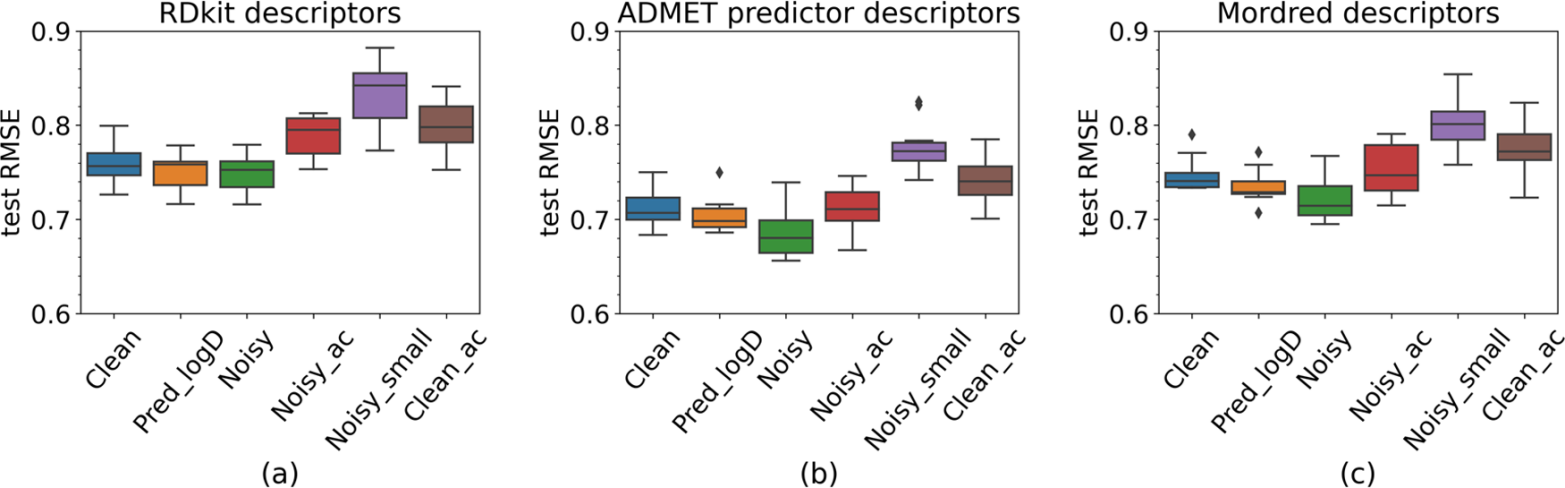
Box plots of the second experiment RMSE for the three descriptor sets: (a) RDKit d, (b) ADMET predictor, and (c) Mordred.

**Figure 6:**
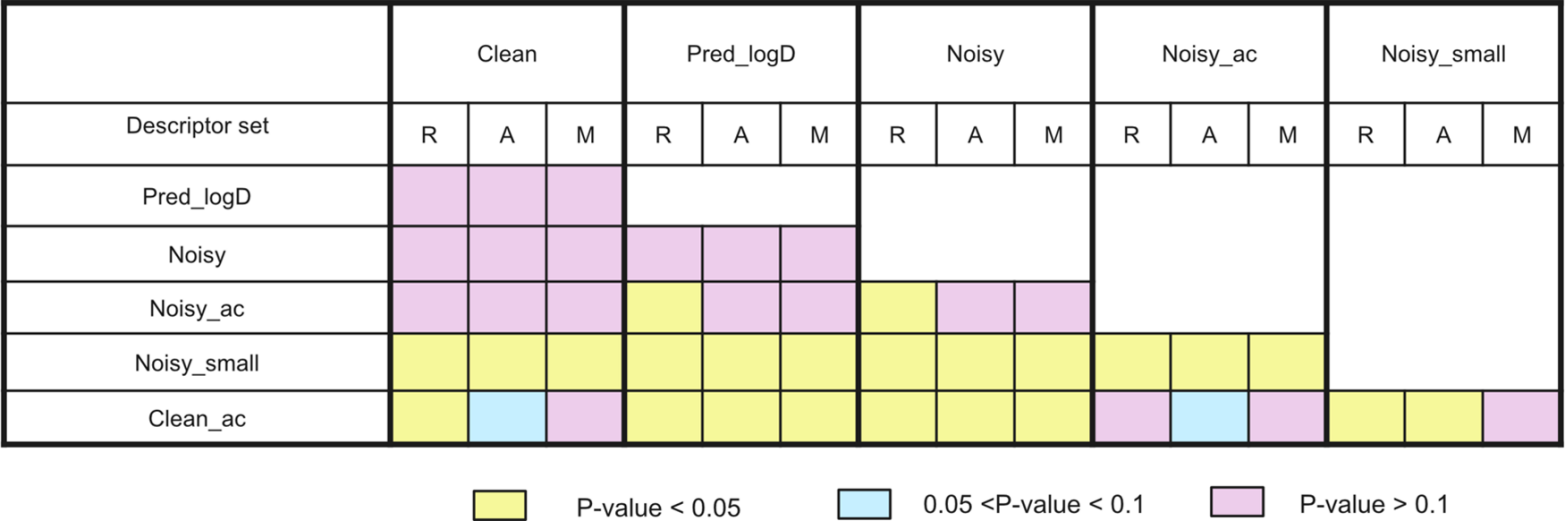
*p*-values are shown in yellow, blue, and pink for each data set pair. Values were calculated with the post hoc Tukey test. R, A, and M represent RDkit, ADMET predictor, and Mordred descriptors, respectively.

Irrespective of the descriptor set used, a general pattern emerged for the models’ performances (Table 3 and Figure 5). Models trained on **Clean**, **Pred_logD**, and **Noisy** were comparable in quality, with **Noisy** having the lowest RMSE and highest *R*^2^. Models trained with **Noisy_ac** and **Clean_ac** were worse than the previous four models, but the differences were not statistically significantly different (Figure 6). **Noisy_small** showed the worst performance.

**Table 3:** RMSE for the Three Descriptor Sets: RDKit, ADMET, and Mordred^a^

RDKit	Clean	Pred_logD	Clean_ac	Noisy	Noisy_ac	Noisy_small
RMSE	0.76(0.02)	0.75(0.02)	0.80(0.03)	0.75(0.02)	0.78(0.02)	0.83(0.03)
*R*^2^	0.41(0.04)	0.41(0.04)	0.34(0.04)	0.41(0.03)	0.34(0.03)	0.29(0.04)
%log *S* ± 0.7	65.7(2.2)	67.1(2.1)	65.2(2.9)	66.4(2.6)	65.5(2.3)	61.3(2.6)

ADMET predictor	Clean	Pred_logD	Clean_ac	Noisy	Noisy_ac	Noisy_small
RMSE	0.71(0.02)	0.71(0.02)	0.74(0.03)	0.69(0.03)	0.71(0.02)	0.78(0.03)
*R*^2^	0.47(0.04)	0.49(0.03)	0.44(0.03)	0.50(0.03)	0.47(0.03)	0.38(0.03)
%Log *S* ± 0.7	67.7(1.6)	69.2(1.4)	68.3(2.8)	70.8(2.2)	69.8(1.7)	64.5(2.4)

Mordred	Clean	Pred_logD	Clean_ac	Noisy	Noisy_ac_	Noisy_small
RMSE	0.75(0.02)	0.73(0.02)	0.77(0.03)	0.72(0.03)	0.75(0.03)	0.80(0.03)
*R*^2^	0.42(0.04)	0.45(0.03)	0.38(0.04)	0.45(0.02)	0.40(0.03)	0.34(0.03)
%log *S* ± 0.7	65.1(1.8)	66.4(1.7)	66.6(2.8)	67.0(2.5)	67.0(2.3)	61.3(2.3)

a Values in parentheses are standard deviations.

These results differ slightly from those in the nested cross-validation evaluation experiment (Figure 4). The **Noisy** and **Noisy_ac** models performed similarly to the **Clean** model when they were evaluated with the clean intrinsic test sets. This indicates that the test set should be carefully chosen as it can impact model ranking. This result agrees with the finding^36^ in the second solubility, challenge where model performance on the “tight” (high-quality) test set was better than on the “loose” (low-quality) test set. The result is also in line with the claim that the observed model performance cannot exceed the internal error of the test set.^38^

## Discussion

### Noise in the Solubility Data Set

During data exploration and data processing, several sources of noise were identified in the solubility data set. To minimize cost in the assay, Polarized Light Microscopy is used to qualitatively evaluate the solids after solubility assessment for the presence of birefringence. While this approach maintains the high-throughput nature of the assay in support of discovery, the disadvantage is that the birefringence cannot be used to determine if the solid is a pure phase or a possible mixture of forms without embarking on costly and time-consuming advanced solid-state analyses. The algorithm used based on log *D* to extract intrinsic solubility assumes that a compound is exclusively acidic or basic, which might also contribute to noise in the intrinsic solubility data sets. Another source of noise comes from the analytical variability. Even though a final quality check for this was implemented, it cannot guarantee the complete removal of this type of noise. Despite these deficiencies, a model with a prediction error equal to the internal error of the in vitro assay is sufficient for early-stage drug development. A quality analysis of the assay was conducted on the compounds for which duplicate measurements were made. There was less variability for high-solubility compounds than for low-soluble compounds. Specifically, 76% of the latter had less than a 5-fold solubility difference. However, since only a minority of the compounds had duplicate measurements, the true error of the assay is difficult to pinpoint. In comparison, our optimal RF model showed almost 70% of the predictions on unseen compounds to fall within 5-fold of the actual solubility (Table 3), indicating that there is still room for improvement.

### Use of Predicted log *D* Values to Extract Intrinsic Solubility

**Clean** and **Pred_logD** compared experimentally determined versus predicted log *D* values to identify the intrinsic solubility. No significant difference emerged from the statistical test (Figure 6), suggesting that log *D* values can be effectively used during the data extraction and training. This can expedite model development and address instances for which experimental log *D* values are unavailable.

### Impact of Analytical Variability in Intrinsic Solubility Prediction

**Clean** and **Noisy_small** were the same size as each other but differed in their noise levels. **Clean** was significantly better (*p* < 0.05), showing that data quality contributes to improved performance of the intrinsic solubility model when the data size is similar (Figure 6).

### Impact of Data Size on Intrinsic Solubility Prediction

The impact of the data size was tested by comparing **Noisy** and **Noisy_small**. Both contained the same level of analytical variability, but **Noisy** contained 3 times more compounds. The performance of **Noisy** was similar (*p* > 0.05) to **Clean** but significantly better than **Noisy_small** (*p* < 0.05). Thus, by increasing data size, the model can overcome analytical variability and give relatively good predictions for all three descriptor sets, similar to those of a smaller high-quality data set.

### Impact of Analytical Variability and Data Size on Intrinsic Solubility Prediction

**Figure 7:**
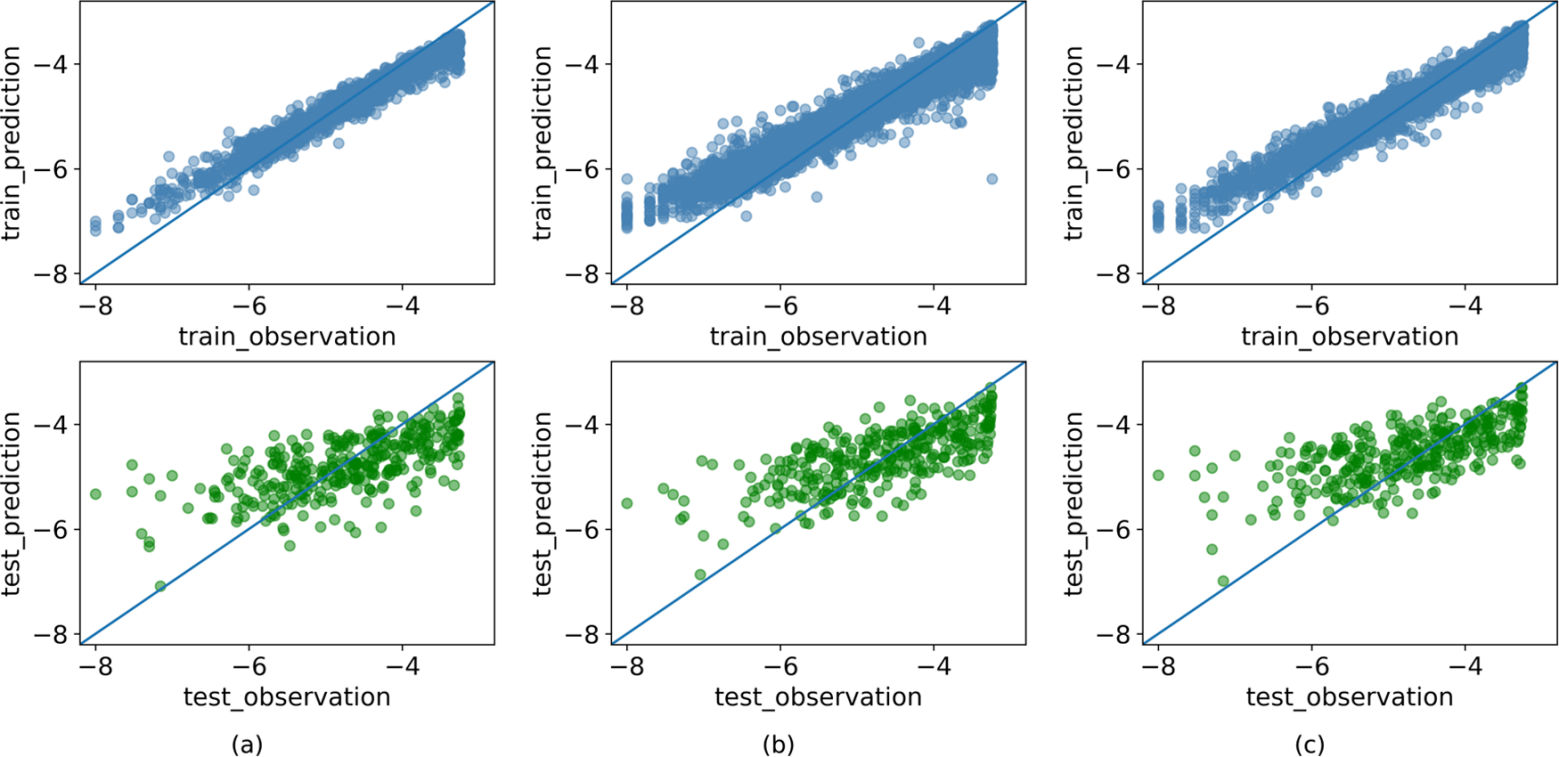
Scatter plots of observed (experimental) and predicted values for training and test sets of models: (a) **Clean**, (b) **Noisy**, and (c) **Clean_ac**.

We also investigated why a model trained with a larger, noisier data set (**Noisy**) performed similarly to one trained with the smaller **Clean**. Scatter plots of true and predicted solubilities were generated from models trained with ADMET predictor descriptors on a randomly selected test set from **Clean** (Figure 7a,b). Both models exhibited notable bias in predicting low- (0.1–1 μM) and high-soluble compounds (100–600 μM). However, predictions made by the **Noisy** model deviated less from the identity line in the high-solubility range, giving better predictions within that region.

**Figure 8:**
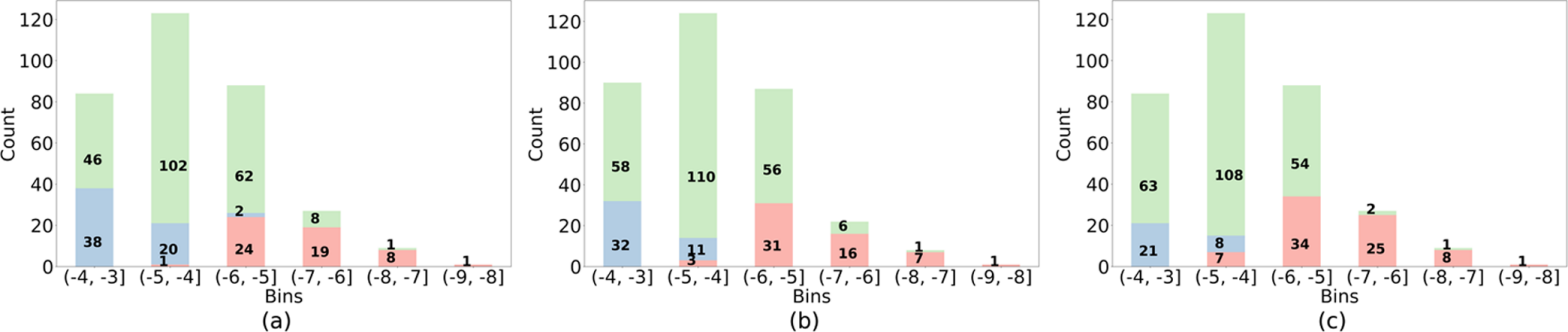
Number of compounds for which solubility was overpredicted (pink), underpredicted (blue), or log *S* ± 0.7 (green). Models: (a) **Clean**, (b) **Noisy, and** (c) **Clean_ac**.

To quantify the number of overpredicted and underpredicted compounds in the test set predictions, the data were binned into six groups. Overpredicted (Predicted log *S* – True log *S* > 0.7) and underpredicted (Predicted log *S* – True log *S* < −0.7) compounds were counted for each bin (Figure 8). Both models tended to overpredict the solubility in the low-solubility range and underpredict it in the high one. There were 53 over- and 60 underpredicted compounds in the test set prediction for **Clean**, and 58 respectively 43 for **Noisy** (Figure 8a,b). The slightly better prediction of **Noisy** for the high-solubility compounds might be because it contains more high-soluble compounds in the training data set (Figure S1b); this potentially helps the model in learning and predicting high-solubility compounds.

### Impact of Solubility Data Combined with Amorphous Solid Residue on Intrinsic Solubility Prediction

The impact of including amorphous solid residues in the training data set was investigated through models built with **Clean_ac**. The model trained with the **Clean_ac** data set was systematically worse than the models trained with **Pred_logD** and **Noisy** (*p* < 0.05, Figure 6).

The results from **Clean_ac** and **Clean** depended on the descriptor sets used. For RDKit descriptors, the p-value was <0.05, meaning model **Clean** was significantly better than model **Clean_ac**. For models trained with ADMET predictor descriptors, the p-value was between 0.05 and 0.1, and <0.1 for models trained with Mordred. Thus, the difference in performance was not statistically significant. However, when comparing the performance of Clean_ac with Pred_logD and Noisy (performing similarly to the Clean data set), a uniform statistical conclusion is valid for all descriptors (*p* < 0.05), indicating a worse performance of model Clean_ac.

Even though, according to the exact numerical p-values, there is insufficient evidence rejecting the null hypothesis when comparing model Clean_ac to model Clean for two descriptor sets, a clear trend across all three descriptor sets can be observed: Clean_ac performs worse than most of the best-performing models. This observation conforms with expectation, as amorphous solid residues tend to increase apparent solubility.

From the pharmaceutical science perspective, the results are also as expected; adding amorphous solids to the data set is unlikely to improve the performance of the model. However, many large data sets in the literature about solubility models are derived from incomplete solubility assays and often omit solid-state testing. Details about the protocol and strict filtering are essential before model development, and awareness of this is important.

### Impact of Solubility Data with Included Amorphous Solid Residue together with Analytical Variability on Intrinsic Solubility Prediction

**Noisy_ac** was the largest data set, containing both analytical variability and noise introduced by including amorphous solid residues in the training set. Even though the difference was not statistically significant between the worse performance of **Noisy_ac** compared to **Clean** and **Noisy**, a clear trend can be perceived (Figure 5 and Table 3). This indicates the inclusion of more data points with amorphous solubility and associated analytical variability will decrease the model performance.

### Impact of Combined Data Set Quality and Size

With increased data size, the random forest model could overcome analytical variability, but not when the training data set contained noise introduced by amorphous solid residues. There is a significant difference between the performances of **Clean_ac** and **Noisy** (Figure 6). This suggests that the two types of noise carry different information about the systems and therefore have different impacts on model performance.

In the comparison of the observed solubility and the solubility predictions (Figure 7b,c), the scatter plots show that the predictions of **Clean_ac** deviate more from the identity line in the lower solubility range than those for **Noisy**. Calculations (Figure 8b,c) show that the model **Noisy** more or less over- and underpredicted the same number of compounds (58 respectively 43). This is in contrast to **Clean_ac**, where the number of solubility overpredictions was much higher than the underpredictions (75 vs 28). The reason might be that amorphous solid residues result in higher solubility compared to crystalline forms. Thus, the inclusion of amorphous residues in a training data set induces a systematic positive bias, which, in turn, leads to a positive bias in the model predictions. Analytical variability, on the other hand, is not biased toward positivity or negativity in the data set. Increasing the size of this data set may even out the noise. This explains why, with a larger data size, **Noisy** gave comparable results to **Clean**. In contrast, **Clean_ac** also had a larger data set than the **Clean** data set but could not overcome the errors. Thus, analytical variability and amorphous noise impact model prediction differently.

### Model Prediction on Second Solubility Challenge Test Set 1

Can a single-source, critically curated training data set enhance intrinsic solubility prediction? To answer this, we tested the optimal models **Clean** and **Noisy** (trained with ADMET predictor descriptors) on the first test set from the second solubility challenge. This set consists of 100 drug-like molecules, with the average intrinsic solubility of each compound calculated from at least three laboratories. The intrinsic solubilities in the data set range (log *S*) from −6.79 to −1.18, with a mean of −4.03, and the average standard deviation is reported to be 0.17 log. Since the intrinsic solubilities in our training data are all below 600 μM, and caution is required when extrapolating machine learning beyond the training data, we removed compounds with intrinsic solubility exceeding −3.22 from the test set. This resulted in 79 compounds being included. The RMSE and *R*^2^ for model **Clean** were found to be 0.74 and 0.31 and 0.72 and 0.34 for model **Noisy**, respectively. The log *S* ± 0.5% value for model **Clean** was 46 and 48% for model **Noisy**. Our models thus achieved the lowest RMSE and top rankings in log *S* ± 0.5% compared to the second solubility challenge. Further improvements might be possible with more sophisticated models to be explored in future studies.

## Conclusions

The effect of data quality and quantity on predictions of intrinsic solubility was investigated in this study. With the advantage of the single-source, large, and high-quality Johnson and Johnson in-house data set, a novel method was implemented to separate different types of noises into different data sets. This enabled an opportunity to investigate how these noises alone impact intrinsic solubility prediction and also how data size interacts with them to affect model performance. Results suggested that when data sizes are similar, the data with higher quality is preferable. Performance decreases caused by analytical variability can be overcome by increasing the data size. However, caution is required when selecting the type of noise to further increase the size of the data set. When the noise is not random but includes bias like the inclusion of data points linked to an amorphous solid residue resulting in a positive systematic error, the quality of the model does not further improve. Finally, two top-performing models were tested with the test set of the second solubility challenge. The results demonstrated that a single-source, critically curated data set enhances the prediction of intrinsic solubility and offers a distinct advantage over the current models based on multiple data sets.

## Supporting Information Available

Boxplot of the six generated intrinsic solubility data sets; descriptors used; hyperparameter tuning range and optimal hyperparameters; and model interpretation with SHAP (PDF)
