# Supplementary material for: Effect of Data Quality and Data Quantity on the Estimation of Intrinsic Solubility: Analysis Based on a Single-Source Data Set
Source: Mol Pharm. 2024 Sep 13;21(10):5261–71. doi: 10.1021/acs.molpharmaceut.4c00685 (PMC11462503; doi:10.1021/acs.molpharmaceut.4c00685)
Supplement: Supplementary file 1 — mp4c00685_si_001.pdf [file mp4c00685_si_001.pdf]

Supporting information

The effect of data quality and data quantity on the estimation of intrinsic solubility - analysis based on single–source dataset

Jiaxi Zhao[1], Eline Hermans[2], Kia Sepassi[3], Christophe Tistaert[2], Christel A. S. Bergström[1], Mazen Ahmad[4], Per Larsson[1*]

[1]Department of Pharmacy, Uppsala University, 751 23 Uppsala, Sweden

[2] Pharmaceutical & Material Sciences, Janssen Pharmaceutica NV, B-2340 Beerse, Belgium

[3]Discovery Pharmaceutics, Janssen Research & Development, LLC, La Jolla, CA 92121, USA

[4] In-silico discovery, Janssen Pharmaceutica NV, B-2340 Beerse, Belgium

[*]Email: per.r.larsson@uu.se

Contents

1. Data exploration 3
2. Descriptors: 3
   2.1 RDkit descriptors: 3
   2.2 ADMET predictor descriptors 3
   2.3 Mordred descriptors 4
3. Hyperparameters: 4
4. Model interpretation with SHAP 5
5. Reference 5

1. Data exploration

From Fig S1 (a), about 60%-75% of compounds in all six datasets have solubility below 100 μM, and in most of the datasets compounds with high solubility values could be considered to be outliers. Fig. S1 (b) present the percentiles for log S, and outliers are compounds with low solubility.

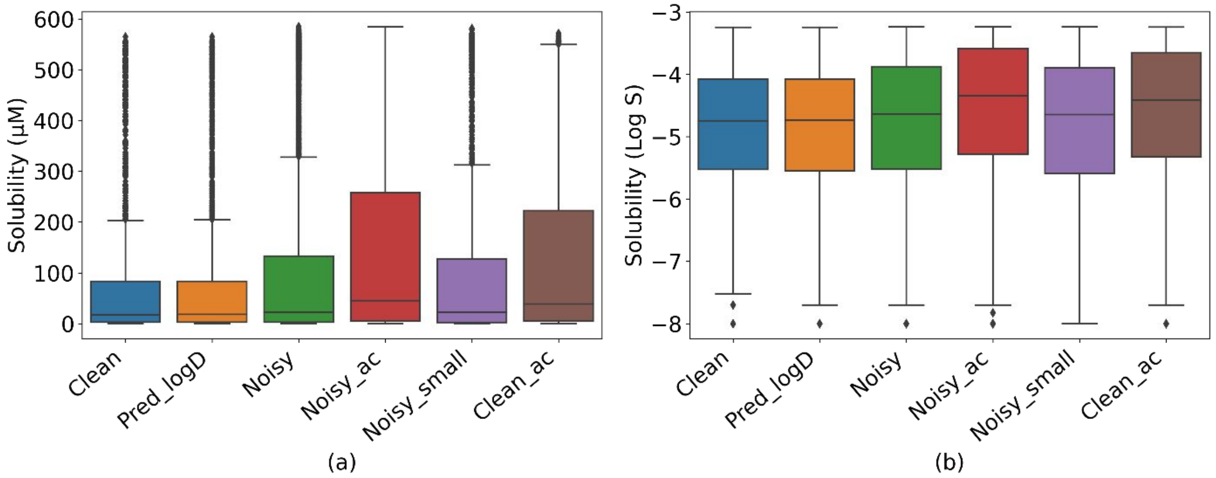

Figure S1. Boxplots of the six generated intrinsic solubility datasets (a) with unit μM (b) log S

2. Descriptors:

2.1 RDkit descriptors:

Molecular weight (Molwt), Topological polar surface area (TPSA), LogP (MolLogP), Number of hydrogen bond acceptors (NumHAcceptors), Number of Nitrogens and Oxygens (NOCount), number of aliphatic heterocycles (NumAliphaticHeterocycles), number of aliphatic rings (NumAliphaticRings), number of aromatic carbocycles (NumAromaticCarbocycles), number of aromatic heterocycles (NumAromaticHeterocycles), number of aromatic rings (NumAromaticRings), Number of hydrogen bond donors (NumHDonors), Number of Heteroatoms (NumHeteroatoms), Number of Rotatable Bonds (NumRotatableBonds), number of saturated carbocycles (NumSaturatedCarbocycles), number of saturated heterocycles (NumSaturatedHeterocycles), number of saturated rings (NumSaturatedRings), number of rings (RingCount), molecular refractivity (MolMR)

2.2 ADMET predictor descriptors

Descriptors within each ADMET predictor descriptors category listed below are calculated, details of the descriptors can be found in the ADMET predictor manual.

- Constitutional descriptors
  Various aspects of molecular size and uniformity of composition e.g. molecular weight, number of different types of atoms, number of different types of rings
- Topological Indices

Numerical descriptors that characterize molecular shape e.g. First Order Simple Kier-Hall shape descriptor (Kappa1), Zeroth Order Simple Connectivity Index (X0)

- Electrotopological State descriptors
  Descriptors that encode information about molecular topology and electronic structure e.g. Atom-type E-state index for -CH3 groups (SsCH3)
- Charge-based group
  Electronic descriptors for the whole molecule or particular region e.g. Sum of Absolute Values of PEOE Partial Atomic Charges (ABSQ)
- Ionization descriptors
  Descriptors related to ionization e.g. Number of recognized ionizable atom types that are acidic (N_IoAcAt)
- Functional groups
  Number of various functional groups e.g. Number of aliphatic hydroxyl groups (AlHdrxl_-OH)
- Hydrogen Bonding Descriptors
  Descriptors related to hydrogen bonds e.g. Number of Intra-molecular Hydrogen Bonds (IHB)

2.3 Mordred descriptors

All 2D descriptors were calculated, 3D descriptors were not included. Details can be found under Mordred 1.2.1a1 documentation[1].

3. Hyperparameters:

Details for specific hyperparameters for LASSO, Random Forst and SVR can be found in scikit-learn[2]. For XGBoost, LightGBM, and ANN, please check XGBoost documentation[3], lightGBM documentation[4] and Keras documentation[5], respectively.

Model	Hyperparameter range	Optimal hyperparameters
LASSO	'alpha': (0.0, 20.0)	alpha: 0.0009
Random Forest	'max_depth': 10, 100<br>'n_estimators': 150,500<br>'min_samples_split': 3,10<br>'max_samples': .6,.98<br>'max_features': ['sqrt', 'log2']	max_depth: 76<br>n_estimators: 477<br>min_sample_split : 3<br>max_samples: 0.87<br>max_features: sqrt
SVR	'C': 0.1, 10<br>'epsilon': 0.001,2<br>'kernel': ['linear', 'rbf']	C: 2.66<br>epsilon: 0.25
XGBoost	'max_depth': 400, 500<br>'n_estimators': 250,300<br>'min_child_weight': 0,10<br>'gamma': 0,5<br>'eta': 0.0, 0.5<br>'subsample': 0.5, 1.0	max_depth: 411<br>n_estimators: 260<br>min_child_weight: 2<br>Gamma: 0<br>Eta: 0.03<br>Subsample: 0.65

	'reg_lambda': 0.0, 10.0	reg_lambda: 6.86
LightGBM	'max_depth': 2, 100<br>'n_estimators': 0,300<br>'num_leaves': 8,4096<br>'learning_rate': 0.0,1.0<br>'min_data_in_leaf': 10, 1000<br>'min_gain_to_split': 0, 15	max_depth: 49<br>n_estimators: 112<br>num_leaves: 2631<br>Learning_rate: 0.37<br>Min_data_in_leaf: 14<br>Min_gain_to_split: 1
ANN	'hidden_layer_sizes_1': 2,124<br>'hidden_layer_sizes_2': 2,124<br>'learning_rate_init': 1e-5, 1e-1	hidden_layer_sizes_1: 115<br>hidden_layer_sizes_2: 2<br>learning_rate_init: 0.003

4. Model interpretation with SHAP

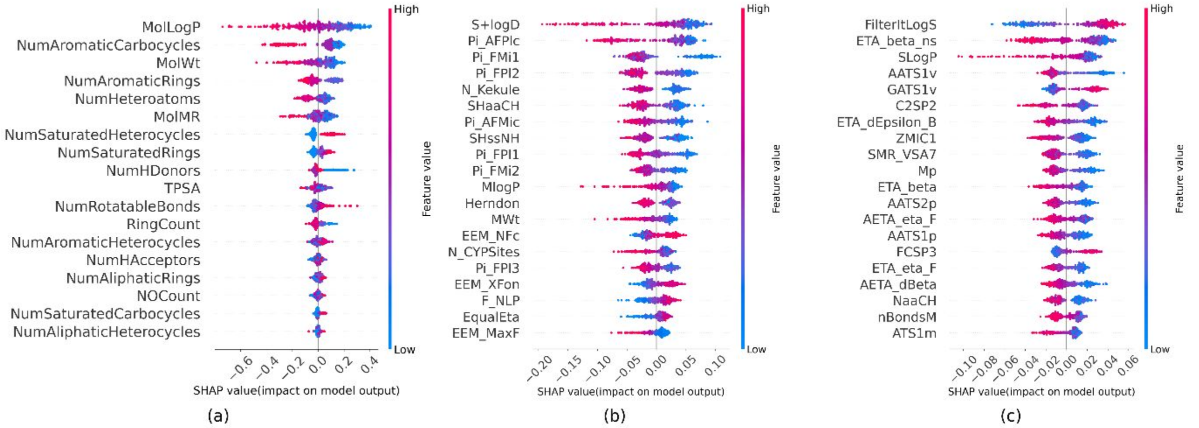

Figure S3. Top contributing features based on SHAP calculation for the three models trained with prediction descriptors on **Clean**: (a) RDKit (b) ADMET (c) Mordred. Each dot represents a specific compound, Red indicates high values and blue low. Dots appear on the left side of the vertical line, contribute negatively to the solubility prediction.

To understand the effect of the features, SHAP analysis was utilized to sort features based on their contribution to the model performance (Fig S3). Molecular weight, log P appears in the top 10 contributing features in all three datasets, which aligns with the earlier findings in the field.

5. Reference

1. Descriptor List — mordred 1.2.1a1 documentation. Accessed July 26, 2024. https://mordred-descriptor.github.io/documentation/master/descriptors.html

2. scikit-learn: machine learning in Python — scikit-learn 1.5.1 documentation. Accessed July 27, 2024. https://scikit-learn.org/stable/

3. XGBoost Python Package — xgboost 2.1.0 documentation. Accessed July 27, 2024. https://xgboost.readthedocs.io/en/stable/python/
4. Welcome to LightGBM's documentation! — LightGBM 4.5.0 documentation. Accessed July 27, 2024. https://lightgbm.readthedocs.io/en/latest/index.html
5. Keras 3 API documentation. Accessed July 27, 2024. https://keras.io/api/
